# Spontaneous summation or numerosity-selective coding?

**DOI:** 10.3389/fnhum.2013.00886

**Published:** 2013-12-19

**Authors:** Qi Chen, Tom Verguts

**Affiliations:** ^1^Center for Studies of Psychological Application and School of Psychology, South China Normal UniversityGuangzhou, China; ^2^Department of Experimental Psychology, Ghent UniversityGhent, Belgium

**Keywords:** numerical cognition, computational modeling, single-unit recording

## Introduction

A key debate in numerical cognition concerns the neural code for number representation (e.g., Nieder and Merten, 2007; Roggeman et al., 2007; Viswanathan and Nieder, 2013). One idea is that individual neurons are tuned to individual numbers, with decreasing response to numbers with increasing distance (numerosity-selective coding or labeled-line coding). An alternative, more implicit way of representing number is by summation coding. Here, individual neurons fire either monotonically stronger or weaker to increasing number. The number can then be decoded from the pooled cell activity.

The computational properties of both coding types have been studied. A summation code but not a numerosity-selective code was extracted without number-related training from a visual display in a recent modeling study (Stoianov and Zorzi, 2012). Also, the summation code serves as a precursor for a numerosity-selective code in such models (Dehaene and Changeux, 1993; Verguts and Fias, 2004). Furthermore, each coding type has distinct advantages; summation coding is more suited for smaller-larger (i.e., magnitude) processing, numerosity-selective coding is more efficient for same-different number discrimination (Verguts, 2007).

In a number of papers, Nieder and colleagues demonstrated numerosity-selective coding in macaque monkeys (e.g., Nieder et al., 2002; Nieder and Miller, 2004). However, number was always relevant for the task; in other words, animals were trained on number (e.g., Nieder et al., 2002). The computational modeling work jointly predicts that summation coding is primary and foundational to numerosity-selective coding, and that in the absence of number-relevant training, only summation coding would be observed. Consistently, Roitman et al. (2007) showed that only summation coding was observed in a single-unit recording study in which number was not relevant for solving the task. However, number was relevant for computing the reward at trial offset, so it may still be that number-relevant learning took place during training.

To determine the natural numerical coding system (i.e., without number learning), Viswanathan and Nieder (2013) recorded cells from ventral intraparietal area [VIP, in intraparietal sulcus (IPS)] and from prefrontal cortex (PFC) in two monkeys in a task without number relevance (and hence number learning). They found that neurons in both brain areas responded maximally to a given number (e.g., one neuron responded maximally to 1, another neuron maximally to 2, and so on). They interpret their data as suggesting numerosity-selective coding. They also found that the most frequently preferred numbers for these neurons were numbers 1 and 5, whereas a relatively small set of neurons were classified as tuned to intermediate numbers 2, 3, and 4. However, given the computational primacy of summation coding, we consider the possibility that the authors instead sampled summation coding neurons. Here, we show that the data are consistent with summation coding, and that summation coding can account for subtle and unexplained aspects of the data.

## Methods

We implemented a summation coding scheme. Neurons were positively tuned to number (*f*_+_, positive-slope neurons) or negatively tuned to number (*f*_−_, negative-slope neurons). Response curves followed logarithmic compression (Pearson et al., 2010):

$$f_{+}=a\log n+b+\text{noise}$$

$$f_{-}=-c\log n+d+\text{noise}$$

where *n* is numerosity. Noise was normally distributed with zero mean. PFC neurons are less noisy than posterior area (e.g., IPS) neurons (e.g., O'Reilly, 2006). Hence, we simulated summation coding neurons in PFC (50 positive-slope and 50 negative-slope neurons) with a low level of noise [standard deviation (*SD*) = 2]; and summation coding neurons in VIP (50 positive-slope, 50 negative-slope) with a higher level of noise (*SD* = 4). The specific standard deviation parameters are chosen to be compatible with the data. Parameters *a, b, c*, and *d* were taken from Pearson et al. (2010) who fit these logarithmic curves to neural response data from Roitman et al. (2007) (values 9.01, 40.20, and 5.34, and 54.6, respectively). Importantly, our results do not depend on the specific parameter values of *SD, a, b, c*, and *d*; other parameter values lead to qualitatively similar results as those reported here. The only requirement is that *SD* in VIP is larger than in PFC, which is a well-motivated assumption (O'Reilly, 2006).

Responses to numbers 1 through 5 were recorded for all neurons. Analyses were carried out as reported in Viswanathan and Nieder (2013). In particular, we identified neurons tuned to specific numbers then plotted their responses to numbers 1–5.

**Figure 1:**
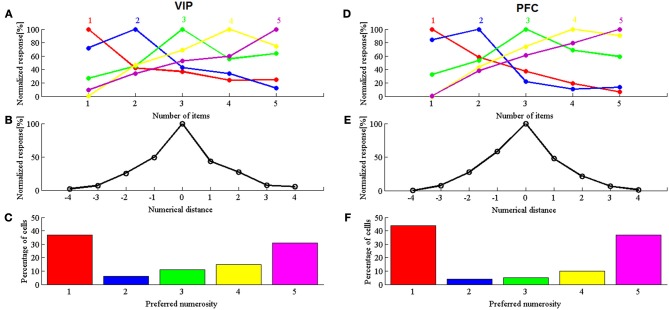
**Simulated results. (A,D)** Normalized response averaged for neurons preferring a given numerosity in area VIP **(A)** and in PFC **(D)**. **(B,E)** simulated normalized discharge rates of neurons in VIP **(B)** and PFC **(E)** plotted against the numerical distance from the preferred numerosity. **(C,F)** simulated frequency distributions of the preferred numerosities for VIP **(C)** and PFC **(F)**, respectively.

Figures 1A,D show responses of neurons tuned to individual numbers in model VIP and PFC, respectively. Figures 1B,E show the aggregate tuning curves. As in Viswanathan and Nieder (2013), numerosity-selective tuning curves emerge from the model even though there is no numerosity-selective coding. This also holds when neurons tuned to 1 and 5 are removed. Figures 1C,F show the distribution of neurons tuned to each specific number. In a completely noiseless system, the distribution would be binary, with 50% of neurons tuned to 1 and 50% of neurons tuned to 5. In a noisy system such as the brain, a significant set of neurons is classified as being tuned to intermediate numbers 2, 3, and 4. This is specifically the case in the (more noisy) VIP (Figure 1C), because a larger standard deviation parameter leads to larger proportions of neurons tuned to intermediated numbers (2, 3, and 4). In addition, the model also accounts for a more subtle aspect of the data. This is the fact that (in the more noisy VIP) there are more neurons tuned to 4 than to 3, and more neurons to 3 than to 2. The reason is that, if an *f*_+_ neuron is misclassified, it is still more likely to be classified as a larger rather than a smaller number. Moreover, misclassification is more likely for *f*_+_ than for *f*_−_ neurons, because of (logarithmic) compression.

## Discussion

Our model is based on summation coding and well-received ideas on number representation (noise, compressed coding). It simulates neural responses in untrained monkeys. It further explains subtle aspects of the tuning distribution that are difficult to interpret from alternative perspectives. In this sense, the report of Viswanathan and Nieder is consistent with the prediction that summation coding is primary and exists without number-relevant training (Verguts and Fias, 2004; Stoianov and Zorzi, 2012).
